# Immunohistochemical prognostic markers of esophageal squamous cell carcinoma: a systematic review

**DOI:** 10.1186/s40880-017-0232-5

**Published:** 2017-08-17

**Authors:** Chunni Wang, Jingnan Wang, Zhaoli Chen, Yibo Gao, Jie He

**Affiliations:** 1 Department of Thoracic Surgery, National Cancer Center/Cancer Hospital, Chinese Academy of Medical Sciences and Peking Union Medical College, Science Building, No.17 Panjiayuan Nanli, Chaoyang District, PO Box 2258, Beijing, 100021 P. R. China; 2 Center for Cancer Precision Medicine, Chinese Academy of Medical Sciences and Peking Union Medical College, Beijing, 100021 P. R. China

**Keywords:** Esophageal squamous cell carcinoma, Prognosis, Survival, Immunohistochemical markers

## Abstract

**Background:**

Esophageal squamous cell carcinoma (ESCC) is an aggressive malignancy, with a high incidence and poor prognosis. In the past several decades, hundreds of proteins have been reported to be associated with the prognosis of ESCC, but none has been widely accepted to guide clinical care. This study aimed to identify proteins with great potential for predicting prognosis of ESCC.

**Methods:**

We conducted a systematic review on immunohistochemical (IHC) prognostic markers of ESCC according to the 2009 Preferred Reporting Items for Systematic Reviews and Meta-Analyses (PRISMA) Guidelines. Literature related to IHC prognostic markers of ESCC were searched from PubMed, Embase, Web of Science, and Cochrane Library until January 30th, 2017. The risk of bias of these original studies was evaluated using the Quality in Prognosis Studies (QUIPS) tool.

**Results:**

We identified 11 emerging IHC markers with reproducible results, including eight markers [epidermal growth factor receptor (EGFR), Cyclin D1, vascular endothelial growth factor (VEGF), Survivin, Podoplanin, Fascin, phosphorylated mammalian target of rapamycin (p-mTOR), and pyruvate kinase M2 (PKM2)] indicating unfavorable prognosis and 3 markers (P27, P16, and E-cadherin) indicating favorable prognosis of ESCC.

**Conclusion:**

Strong evidence supports that these 11 emerging IHC markers or their combinations may be useful in predicting prognosis and aiding personalized therapy decision-making for ESCC patients.

**Electronic supplementary material:**

The online version of this article (doi:10.1186/s40880-017-0232-5) contains supplementary material, which is available to authorized users.

## Background

Esophageal cancer is the sixth leading cause of cancer death and the eighth most common cancer worldwide, with more than 480,000 new cases and 400,000 deaths each year [1]. Although the incidence of esophageal adenocarcinoma is rising in North America and Europe, esophageal squamous cell carcinoma (ESCC) remains the predominant histological type of esophageal cancer worldwide [2]. Surgery alone or in combination with neoadjuvant chemoradiotherapy, adjuvant radiotherapy, and/or adjuvant chemotherapy remains the main curative modality for ESCC. The clinical treatment decision is based mainly on TNM (tumor, node, metastasis) staging [3]. However, given the insidious symptoms, late clinical presentation, and rapid progression of the disease, the prognosis of ESCC remains extremely poor. In China, ESCC remains the fourth leading cause of cancer-related death [4], and the 5-year survival rate of ESCC patients who undergo surgery is only 30%–40% [5].

Better knowledge of patient prognosis would help guide surgery or adjuvant treatment. Molecules identified as critical in carcinogenesis and cancer progression may help classify patients at the same stage into different subgroups in terms of their prognosis, e.g., estrogen receptor (ER) status and human epidermal growth factor receptor-2 (HER2) status in breast cancer patients [6]. Much effort has been made to identify prognostic markers of ESCC. Recently, Chen et al. [7] comprehensively evaluated the prognostic values of copy number variation (CNV), mutations, and relative expression of genes in ESCC. They identified mutations in neurogenic locus notch homolog protein 1 (*NOTCH1*) as well as CNVs in MYB proto-oncogene like 2 (*MYBL2*) and microRNA-4707-5p, and subsequently validated the prognostic values of these genes based on the expression profiles of an independent retrospective ESCC cohort [7]. Many studies have been conducted to evaluate the prognostic values of proteins detected with immunohistochemistry (IHC) in ESCC. Most of these studies were conducted retrospectively, and significant heterogeneity has been noted in the patient populations (regions, races, and disease stages), treatments employed, antibodies used, IHC scoring methods, and length of follow-up. Given these limitations, the prognostic values of most proteins may not be reproducible among different populations. In addition, no IHC biomarker has been accepted into clinical prognostic models in practice, such as the TNM classification for ESCC. Therefore, we conducted a systematic review of the published literature to summarize potential prognostic biomarkers that may be worthy of validation in well-designed, large, prospective trials.

## Materials and methods

### Data source and study selection

This review was conducted according to the 2009 Preferred Reporting Items for Systematic Reviews and Meta-Analyses (PRISMA) Guidelines [8]. We searched the PubMed, Embase, Web of Science, and Cochrane Library with the key phrases “esophageal squamous cell carcinoma OR oesophageal squamous cell carcinoma OR ESCC” AND “prognosis OR prognostic OR outcome OR survival OR recurrence OR relapse OR response” AND “expression” with the search limited to “humans” until January 30th, 2017.

Two investigators (CW and JW) independently screened the retrieved literature by title and abstract for inclusion in the review. If the suitability of an article was uncertain, the full text was assessed. Disagreements were resolved by consensus or reviewed by a third investigator (ZC). The criteria used to determine study eligibility were as follows: (1) a prospective or retrospective cohort with a minimum of 50 patients; (2) assay of primary ESCC specimens; (3) assessment of the expression of target proteins with IHC; (4) analysis of the associations of markers with disease-specific survival (DSS), disease-free survival (DFS), progression-free survival (PFS), or overall survival (OS); and (5) full text available. Studies were excluded when the target proteins were evaluated in less than four independent original studies. Moreover, when overlapping patient cohorts were used to investigate the prognostic value of one marker in multiple studies, the one with a smaller sample size was excluded from the review. Meta-analyses papers on the prognostic value of the protein of interest were considered and included, whereas the original reports involved in those meta-analyses were excluded. The subsequent original reports on the same protein published after the meta-analyses were also reviewed and described in the present systematic review.

We considered the proteins “emerging markers” according to the criteria as follows: (1) more than half of the original studies revealed that the expression of a given protein was significantly associated with prognosis; (2) the independent prognostic significance of the protein was demonstrated by multivariate analysis in 3 or more original studies.

### Data extraction and assessment

Two reviewers (CW and JW) independently extracted data on country, sample size, age, gender, tumor stage, specific proteins, and the results of statistical analyses from the selected original studies. Study quality was assessed using the PRISMA Statement [8]. The Quality in Prognosis Studies (QUIPS) tool [9] was used to evaluate the risk of bias of these original studies. Since all original studies were retrospective studies, they were not evaluated for items b, c, and e of the second domain (study attrition) [9]. Risk of bias was graded as high, moderate, or low according to prompting items.

## Results

### Study selection and study characteristics

**Fig. 1:**
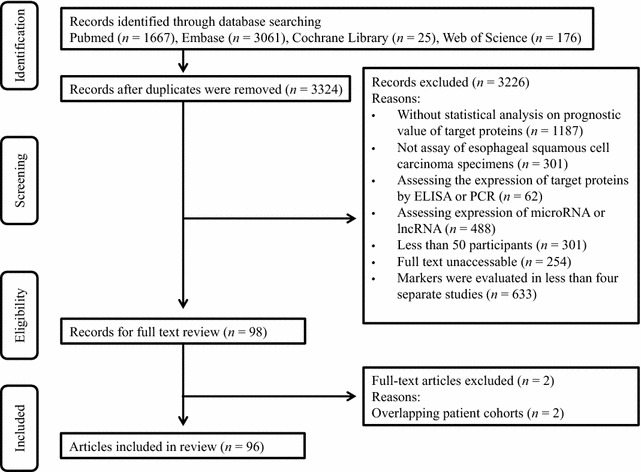
Flowchart of study selection for this systematic review on immunohistochemical prognostic markers of esophageal squamous cell carcinoma. *ELISA* enzyme-linked immunosorbent assay, *PCR* polymerase chain reaction, *lncRNA* long non-coding RNA

Dating to January 30th, 2017, a total of 3324 articles were retrieved from PubMed, Embase, Web of Science, and Cochrane Library as illustrated in Fig. 1. A total of 3226 articles were excluded after reviewing titles and abstracts. Two were excluded after full-text review. Finally, 96 studies, including 14 meta-analyses (Table 1) and 82 original studies (Tables 2, 3, 4, 5, 6, 7) analyzing 30 proteins, were included. The characteristics of the original studies are illustrated in Additional file 1: Table S1. All original studies were conducted retrospectively. The sample size varied between 51 and 590 ESCC patients. More than half (53%–98%) of ESCC patients were men in all original studies. The median age of ESCC patients varied between 52 and 66 years old, notably 9 original studies failed to report a median age [10–18]. The majority of the original studies were conducted in China (50.0%, 41/82) and Japan (35.3%, 29/82).

**Table 1:** Meta-analyses references of the studies on candidate IHC markers for survival in ESCC

Marker	References	Publication period of involved studies	Number of eligible studies	Number of patients	Pooled HR	95% CI
EGFR	Yu et al. [27]	Until Nov, 2010	5	462	1.60	1.05–2.43
	Wang et al. [28]	Until Dec, 2013	13	1150	1.768	1.039–3.007
Cyclin D1	Zhao et al. [40]	Until Apr, 2010	10	1376	1.78	1.49–2.12
	Chen et al. [41]	Until Apr, 2012	12	1295	1.82	1.50–2.20
P21	Chen et al. [41]	Until Apr, 2012	7	683	1.28	0.70–2.33
P27	Chen et al. [41]	Until Apr, 2012	6	478	0.51	0.26–1.00
P53	Chen et al. [41]	Until Apr, 2012	20	2063	1.25	1.03–1.51
Survivin	Chen et al. [41]	Until Apr, 2012	4	295	1.57	0.91–2.69
	Li et al. [58]	Until Mar, 2012	3 (nuclei)	277	1.89	1.45–2.96
			2 (cytoplasm)	113	0.96	0.16–5.69
	Xia et al. [59]	Until Nov, 2014	8	573	1.82	1.43–2.30
VEGF	Chen et al. [41]	Until Apr, 2012	16	1329	1.84	1.45–2.33
	Chen et al. [71]	Until Dec, 2011	26	2043	1.81	1.57–2.10
HIF-1α	Ping et al. [74]	Until Sep, 2013	12	942	1.78	1.41–2.24
	Sun et al. [75]	Until Dec, 2011	16	1261	0.32	0.115–0.887
E-cadherin	Chen et al. [41]	Until Apr, 2012	7	977	0.81	0.64–1.01
	Xu et al. [78]	Until Jun, 2012	9	1129	0.72	0.64–0.83
MTA1	Luo et al. [94]	Until Oct, 2013	4	465	1.86	1.44–2.39
PD-L1	Qu et al. [97]	Until Jul, 2016	7	1350	1.65	0.95–2.85
COX-2	Chen et al. [41]	Until Apr, 2012	4	234	0.96	0.39–2.41
	Li et al. [102]	Until Dec, 2008	12	1167	1.42	1.07–1.90
OCT4	Nagaraja et al. [103]	Until May, 2013	4	539	2.900	1.843–4.565

*IHC* immunohistochemistry, *ESCC* esophageal squamous cell carcinoma, *EGFR* epidermal growth factor receptor, *VEGF* vascular endothelial growth factor, *HIF-1α* hypoxia-inducible factor-1α, *MTA1* metastasis-associated protein 1, *PD-L1* programmed cell death-ligand 1, *COX-2* cyclooxygenase-2, *OCT4* octamer-binding transcription factor 4, *HR* hazard ratio, *CI* confidence interval

**Table 2:** Prognostic markers involved in regulating proliferation in ESCC as reported in original studies

Marker	References	Sample size	Clinical stage	OS			DFS			Analytic methods
				HR	95% CI	*P* value	HR	95% CI	*P* value	
EGFR	Zhang et al. [10]	441	IB-IIIC	1.452	1.137–1.855	0.003	1.351	1.057–1.728	0.016	Cox proportional hazards model
	Zhang et al. [19]	128	II–IV	–	–	>0.05	–	–	–	Log-rank test
	Cao et al. [29]	315	I–IV	1.614	1.027–2.536	0.038	–	–	–	Cox proportional hazards model (univariate)
	Shang et al. [30]	590	I-III	2.652	1.708-4.118	0.00001	–	–	–	Cox proportional hazards model
	Jiang et al. [31]	96	–	–	–	0.007	–	–	0.006	Log-rank test
	Xu et al. [32]	87	I–III	1.728	1.011–2.955	0.046	–	–	–	Cox proportional hazards model
HER2	Zhang et al. [19]	128	II–IV	–	–	>0.05	–	–	–	Log-rank test
	Mimura et al. [33]	66	0–IV	0.92	0.35–2.41	0.861	–	–	–	Cox proportional hazards model,
	Sunpaweravong et al. [34]	55	II–IV	–	–	0.04	–	–	–	Log-rank test
	Zhan et al. [35]	145	I–IV	–	–	0.036	–	–	–	Log-rank test
p-mTOR	Hirashima et al. [36]	143	I–III	2.92	1.48–5.78	0.002	–	–	–	Cox proportional hazards model
	Kim et al. [37]	165	I–IV	1.47	0.92-2.35	0.104	1.67	1.07–2.62	0.025	Cox proportional hazards model
	Li et al. [38]	105	I–IV	–	–	0.022	–	–	0.014	Log-rank test
	Li et al. [39]	77	II–III	2.814	1.553–5.097	0.001	2.438	1.368–4.347	0.003	Cox proportional hazards model
P16	Mathew et al. [11]	50	I–IV	–	–	>0.05	–	–	–	Log-rank test
	Okamoto et al. [20]	86	I–IV	–	–	0.19	–	–	0.14	Cox proportional hazards model (univariate)
	Cao et al. [42]	105	I–III	4.23	1.75–8.54	0.03	2.52	1.12–5.71	0.02	Cox proportional hazards model
	Guan et al. [43]	90	I–IV	0.234	0.086–0.637	0.004	–	–	–	Cox proportional hazards model
	Takeuchi et al. [44]	90	I–III	0.312	–	0.003	–	–	–	Cox proportional hazards model
	Guner et al. [45]	53	I–III	0.410	0.203–0.828	0.013	–	–	–	Cox proportional hazards model (univariate)
	Fujiwara et al. [46]	60	I–IV	0.597	0.287–1.032	0.067	–	–	–	Cox proportional hazards model
P21	Shiozaki et al. [12]	69	I–IV	0.381	0.123–0.995	0.049	–	–	–	Cox proportional hazards model

*ESCC* esophageal squamous cell carcinoma, *EGFR* epidermal growth factor receptor, *HER2* human epidermal growth factor receptor-2, *p-mTOR* phosphorylated mammalian target of rapamycin, *OS* overall survival, *DFS* disease-free survival, *HR* hazard ratio, *CI* confidence interval, *–* no data

**Table 3:** Prognostic markers involved in suppressing growth of ESCC as reported in original studies

Marker	References	Sample size	Clinical stage	OS			DFS			Analytic methods
				HR	95% CI	*P* value	HR	95% CI	*P* value	
Rb	Mathew et al. [11]	50	I–IV	–	–	>0.05	–	–	–	Log-rank test
	Takeuchi et al. [44]	90	I–III	0.218	–	0.11	–	–	–	Log-rank test
	Guner et al. [45]	53	I–IV	0.588	0.255–1.344	0.207	–	–	–	Cox proportional hazards model (univariate)
	Ikeguchi et al. [47]	191	I–IV	0.730	0.472–1.126	0.155	–	–	–	Cox proportional hazards model
	Ikeguchi et al. [48]	107	I–IV	–	–	–	0.769	0.471–1.222	0.257	Cox proportional hazards model
	Nam et al. [49]	51	I–IV	–	–	>0.05	–	–	–	Log-rank test
	Nita et al. [50]	62	I–III	–	–	0.6811	–	–	–	Log-rank test
	Wang et al. [51]	100	I–IV	–	–	>0.05	–	–	–	Log-rank test
P53	Okamoto et al. [20]	86	I–IV	–	–	0.30	–	–	0.55	Log-rank test
	Shang et al. [30]	590	I–III	1.556	1.063–2.277	0.0229	–	–	–	Cox proportional hazards model
	Huang et al. [52]	106	I–IV	0.732	0.531–1.010	0.060	–	–	–	Cox proportional hazards model
	Murata et al. [53]	266	I–IV	–	–	0.62	–	–	0.73	Log-rank test
	Wang et al. [54]	114	II–III	0.800	0.254–3.182	0.597	–	–	–	Cox proportional hazards model

*ESCC* esophageal squamous cell carcinoma, *Rb* retinoblastoma-associated protein, *OS* overall survival, *DFS* disease-free survival, *HR* hazard ratio, *CI* confidence interval, – no data

**Table 4:** Prognostic markers involved in regulating cell apoptosis in ESCC as reported in original studies

Marker	References	Sample size	Clinical stage	OS			DFS			Analytic methods
				HR	95% CI	*P* value	HR	95% CI	*P* value	
MDM2	Mathew et al. [11]	50	I–IV	–	–	>0.05	–	–	–	Log-rank test
	Okamoto et al. [20]	86	I–IV	–	–	0.31	–	–	0.61	Log-rank test
	Nam et al. [49]	51	I–IV	–	–	>0.05	–	–	–	Log-rank test
	Ikeguchi et al. [55]	107	I–IV	2.017	1.098–3.703	0.024	–	–	–	Cox proportional hazards model
	Sun et al. [56]	149	I–IV	1.698	1.054–	0.03	–	–	–	Cox proportional hazards model
	Cheng et al. [57]	119	I–IV	0.168	0.533–1.509	0.682	–	–	–	Cox proportional hazards model
Fas	Chan et al. [60]	58	I–IV	0.639	0.442–0.925	<0.05	–	–	–	Cox proportional hazards model
	Shibakita et al. [61]	106	I–IV	3.26	1.32–8.07	0.0103	–	–	–	Cox proportional hazards model
	Chang et al. [62]	118	I–IV	–	–	>0.05	–	–	–	Log-rank test
	Takikita et al. [63]	313	I–IV	0.79	0.49–1.27	0.32	–	–	–	Cox proportional hazards model
Bax	Ikeguchi et al. [13]	141	I–IV	–	–	>0.05	–	–	–	Cox proportional hazards model
	Guner et al. [45]	53	I–IV	0.474	0.238–0.941	0.0328	–	–	–	Cox proportional hazards model
	Chang et al. [62]	118	I–IV	–	–	>0.05	–	–	–	Cox proportional hazards model
	Sturm et al. [64]	53	I–IV	0.435	0.242–0.862	0.016	–	–	–	Cox proportional hazards model
	Kurabayashi et al. [65]	76	I–IV	–	–	<0.05	–	–	–	Log-rank test
	Natsugoe et al. [66]	111	II–IV	–	–	>0.05	–	–	–	Log-rank test
	Takayama et al. [67]	86	I–IV	0.954	0.517–1.763	0.881	–	–	–	Cox proportional hazards model
	Matsumoto et al. [68]	79	–	–	–	>0.05	–	–	–	Cox proportional hazards model
	Sarbia et al. [69]	172	I–IV	–	–	>0.05	–	–	–	Log-rank test
Bcl-2	Guner et al. [45]	53	I–IV	1.280	0.688–2.382	0.4364	–	–	–	Cox proportional hazards model (univariate)
	Chang et al. [62]	118	I–IV	0.529	0.387–0.978	0.042	–	–	–	Cox proportional hazards model
	Takikita et al. [63]	313	I–IV	1.29	0.52–3.25	0.58	–	–	–	Cox proportional hazards model
	Kurabayashi et al. [65]	76	I–IV	–	–	>0.05	–	–	–	Log-rank test
	Takayama et al. [67]	86	I–IV	1.506	0728–3.115	0.269	–	–	–	Cox proportional hazards model
Bcl-x	Natsugoe et al. [66]	111	II–IV	–	–	>0.05	–	–	–	Log-rank test
	Takayama et al. [67]	86	I–IV	2.441	1.139–5.232	0.022	–	–	–	Cox proportional hazards model
	Matsumoto et al. [68]	79	–	–	–	0.194	–	–	–	Cox proportional hazards model
	Torzewski et al. [70]	172	I–IV	–	–	>0.05	–	–	–	Cox proportional hazards model
Caspase-3	Jiang et al. [14]	64	I–III	–	–	0.007	–	–	–	Cox proportional hazards model
	Wang et al. [21]	122	–	0.584	0.370–0.921	0.021	–	–	–	Cox proportional hazards model
	Chang et al. [62]	118	I–IV	–	–	>0.05	–	–	–	Cox proportional hazards model
	Kurabayashi et al. [65]	76	I–IV	–	–	>0.05	–	–	–	Log-rank test

No original studies on the prognostic significance of Survivin in ESCC were reported after the meta-analyses. Therefore, Survivin is not listed

*ESCC* esophageal squamous cell carcinoma, *MDM2* murine double minute gene 2, *OS* overall survival, *DFS* disease-free survival, *HR* hazard ratio, *CI* confidence interval, *–* no data

**Table 5:** Prognostic markers involved in regulating angiogenesis in ESCC as reported in original studies

Marker	References	Sample size	Clinical stage	OS			DFS			Analytic methods
				HR	95% CI	*P* value	HR	95% CI	*P* value	
VEGF	Tao et al. [15]	90	–	0.027	0.009–0.079	<0.001	–	–	–	Cox proportional hazards model
	Huang et al. [52]	106	I–IV	1.214	0.639–2.305	0.554	–	–	–	Cox proportional hazards model
	Hou et al. [72]	483	I–III	1.864	1.055–3.294	0.032	2.077	1.265–3.411	<0.01	Cox proportional hazards model
	Omoto et al. [73]	119	–	1.237	0.919–1.649	0.157	–	–	–	Cox proportional hazards model
HIF-1α	Shirakawa et al. [76]	229	I–IV	–	–	>0.05	–	–	–	Log-rank test
	Zhang et al. [77]	136	I–IV	1.297	0.856–1.964	0.220	1.234	0.794–1.920	0.350	Cox proportional hazards model

*ESCC* esophageal squamous cell carcinoma, *VEGF* vascular endothelial growth factor, *HIF-1α* hypoxia-inducible factor-1α, *OS* overall survival, *DFS* disease-free survival, *HR* hazard ratio, *CI* confidence interval, – no data

**Table 6:** Prognostic markers involved in activating invasion and metastasis of ESCC as reported in original studies

Marker	References	Sample size	Clinical stage	OS			DFS			Analytic methods
				HR	95% CI	*P* value	HR	95% CI	*P* value	
E-cadherin	Ozawa et al. [79]	83	I–IV	–	–	0.022	–	–	0.003	Log-rank test
α-catenin	Nakanishi et al. [22]	96	I–III	0.741	0.160–3.450	0.70	–	–	–	Cox proportional hazards model
	Nair et al. [26]	100	I–III	–	–	>0.05	–	–	–	Log-rank test
	Setoyama et al. [80]	205	I–IV	0.468	0.314–0.664	<0.001	–	–	–	Cox proportional hazards model
	Lin et al. [81]	62	I–III	–	–	>0.05	–	–	–	Log-rank test
β-catenin	Lv et al. [23]	70	I–IV	0.034	0.009–0.144	0.002	–	–	–	Cox proportional hazards model
	Nair et al. [26]	100	I–III	–	–	>0.05	–	–	–	Log-rank test
	Chang et al. [62]	118	I–IV	–	–	>0.05	–	–	–	Log-rank test
	Lin et al. [81]	62	I–III	–	–	>0.05	–	–	–	Log-rank test
	Situ et al. [82]	227	II	1.642	1.159–2.327	0.005	–	–	–	Cox proportional hazards model
	Hsu et al. [83]	68	I–V	0.433	0.244–0.765	0.004 (membrane)	–	–	–	Cox proportional hazards model
				–	–	0.821 (cytoplasm)	–	–	–	
	Zhao et al. [84]	106	I–IV	–	–	>0.05	–	–	–	Log-rank test
	Li et al. [85]	128	I–IV	–	–	0.569	–	–	0.503	Log-rank test
	Deng et al. [86]	100	–	–	–	0.872	–	–	–	Log-rank test
Podoplanin	Tong et al. [16]	56	I–IV	13.83	3.06–62.43	0.001	–	–	–	Cox proportional hazards model
	Chao et al. [87]	113	II–IV	–	–	–	1.951	1.231–3.090	0.004	Cox proportional hazards model
	Nakashima et al. [88]	101	I–IV	2.16	1.05–4.65	0.036	–	–	–	Cox proportional hazards model
	Tanaka et al. [89]	139	I–III	3.084	1.543–6.164	0.001	–	–	–	Cox proportional hazards model
	Rahadiani et al. [90]	61	I–IV	1.926	1.085–3.421	0.0253	1.931	1.087–3.431	0.0249	Cox proportional hazards model (univariate)
Fascin	Cao et al. [29]	315	I–IV	1.749	1.065–2.873	0.027	–	–	–	Cox proportional hazards model
	Hashimoto et al. [91]	200	I–IV	1.79	1.15–2.77	0.0094	–	–	–	Cox proportional hazards model
	Zhao et al. [92]	254	I–IV	1.604	1.145–2.248	0.006	–	–	–	Cox proportional hazards model
	Takikita et al. [93]	257	I–IV	1.06	0.76–1.48	0.72	–	–	–	Cox proportional hazards model

No original studies on the prognostic significance of metastasis-associated protein 1 (MTA1) in ESCC were reported after the meta-analyses. Therefore, MTA1 is not listed

*ESCC* esophageal squamous cell carcinoma; *OS* overall survival, *DFS* disease-free survival, *HR* hazard ratio, *CI* confidence interval, *–* no data

**Table 7:** Prognostic markers involved in other aspects of ESCC as reported in original studies

Marker	References	Sample size	Clinical stage	OS			DFS			Analytic methods
				HR	95% CI	*P* value	HR	95% CI	*P* value	
PKM2	Zhan et al. [17]	210	I–IV	1.748	1.277–2.395	<0.001	–	–	–	Cox proportional hazards model
	Li et al. [24]	141	I–IV	1.214	0.728–2.026	0.458	–	–	–	Cox proportional hazards model
	Zhang et al. [95]	86	I–IV	2.358	1.156–4.812	0.018	–	–	–	Cox proportional hazards model
	Fukuda et al. [96]	205	I–IV	1.850	1.200–2.780	0.0189	–	–	–	Cox proportional hazards model
CXCR4	Gockel et al. [25]	53	I–III	1.472	0.836–2.593	0.181	–	–	–	Cox proportional hazards model
	Zhang et al. [98]	136	I–IV	1.612	1.072–2.425	0.022	1.708	1.126–2.591	0.012	Cox proportional hazards model
	Lu et al. [99]	127	I–III	1.720	0.749–3.928	0.202	1.497	0.659–3.399	0.335	Cox proportional hazards model
	Qi et al. [100]	60	–	–	–	0.001	–	–	–	Log-rank test
	Sasaki et al. [101]	214	I–IV	–	–	0.4	–	–	0.3	Log-rank test
MLH1	Tzao et al. [18]	60	I–IV	–	–	0.18	–	–	–	Log-rank test
	Kishi et al. [104]	156	I–IV	2.020	1.146–4.231	0.018	–	–	–	Cox proportional hazards model
	Uehara et al. [105]	122	I–IV	–	–	0.0043	–	–	–	Log-rank test

*ESCC* esophageal squamous cell carcinoma, *PKM2* pyruvate kinase M2, *CXCR4* C-X-C chemokine receptor type 4, *MLH1* mut-L-homologon-1, *OS* overall survival, *DFS* disease-free survival, *HR* hazard ratio, *CI* confidence interval, *–* no data

### Quality assessment

Quality assessment was conducted using the QUIPS tool [9]. Approximately one-third (39.0%, 32/82) of these original studies showed a moderate risk of bias for domain 1 (“Study Participation”), primarily due to small participation cohorts (Additional file 2: Table S2). For domain 2 (“Study Attrition”), 73 original studies showed a low risk of bias because of the high follow-up rate for study participants. Seven original studies had moderate bias in domain 2 due to missing data on participants that were lost to follow-up [19–25]. There was a high risk of bias in domain 2 in 2 studies because of high loss to follow-up rates (50 and 23%) [11, 26]. All original studies provided clear description of prognostic factors and clear definitions of outcomes and thus were all ranked as having a low risk of bias for domain 3 (“Prognostic Factor Measurement”) and domain 4 (“Outcome Measurement”). Moreover, 25 of the 82 original studies conducted only log-rank analyses, without multivariate Cox analysis. These original studies were ranked as having a moderate risk of bias for domain 5 (“Statistical Analysis and Reporting”).

### Associations between proliferation-related markers and prognosis of ESCC patients

Seven markers are involved in proliferation-sustaining signalling in ESCC, including epidermal growth factor receptor (EGFR), human epidermal growth factor receptor-2 (HER2), phosphorylated mammalian target of rapamycin (p-mTOR), Cyclin D1, P16, P21, and P27 (Tables 1, 2).

#### EGFR

Yu et al. [27] systematically reviewed 9 original studies published between 1991 and 2010, of which five concerned OS and EGFR overexpression. Significant associations between EGFR overexpression and lymph node status and differentiation grade were noted. Four of the 5 original studies revealed prognostic significance of EGFR overexpression. Meta-analysis demonstrated that EGFR overexpression was associated with short OS.

Recently, Wang et al. [28] conducted a meta-analysis of original studies published before December 2013 that produced the same conclusion. Five original studies published after December 2013 demonstrated a significant association between EGFR overexpression and poor prognosis [10, 29–32]. Of note, 3 original studies indicated that EGFR overexpression may be an independent prognostic marker in ESCC patients [10, 30, 32]. Overall, strong evidence has suggested that the strength of this significance warrants confirmation in clinical trials with more homogeneous and well-defined populations.

#### HER2

Although 3 original studies of HER2 in ESCC indicated that patients without HER2 protein expression exhibited a higher survival rate than those with HER2 expression [33–35], no evidence suggests that HER2 expression may be an independent prognostic predictor in patients with ESCC.

#### p-mTOR

Four original studies investigated mTOR activation status and its prognostic significance in ESCC [36–39]. Approximately 50% of the patients in these original studies were p-mTOR-positive. All the 4 original studies indicated that a high level of p-mTOR was associated with unfavorable prognosis. Moreover, the independent prognostic value of p-mTOR in ESCC was demonstrated in 2 original studies [36, 39].

#### Cyclin D1

The prognostic significance of Cyclin D1 in ESCC has been extensively studied. Zhao et al. [40] conducted a meta-analysis of 10 original studies regarding the prognostic significance of Cyclin D1 expression in ESCC published before April 2010 and comprising 1376 patients. Of these 10 original studies, eight identified Cyclin D1 expression as an independent prognostic factor of ESCC. The pooled hazard ratio (HR) for Cyclin D1 expression was 1.78, indicating that the overexpression of Cyclin D1 was significantly associated with poor prognosis of ESCC patients. In 2013, Chen et al. [41] conducted a systematic review and meta-analysis of tumor biomarkers in predicting prognosis in esophageal cancer. Twelve studies comprising 1295 ESCC patients were enrolled to evaluate the prognostic significance of Cyclin D1 expression in ESCC, and two evaluated the expression of Cyclin D1 using polymerase chain reaction assay (PCR) instead of IHC. The pooled HR was 1.82, which is very consistent with the results of previous studies.

#### P16

The association of P16 expression with favorable prognosis in ESCC was demonstrated in 3 separate original studies with multivariate analysis [42–44]; two studies demonstrated the prognostic value of P16 expression only with univariate analysis [45, 46]. However, no prognostic significance of P16 was shown in two other original studies [11, 20]. Notably, P16 expression combined with other markers may serve as a better prognostic factor in ESCC patients. In the study conducted by Mathew et al. [11], univariate analysis revealed that pRb−/P16−/P21− (*P* = 0.03) and P53+/P16−/pRb− (*P* = 0.02) were prognostic indicators for short OS. In a subsequent original study, the OS rate of patients with P16+/VEGF− was significantly higher than that of other patient groups [44].

#### P21

According to the meta-analysis performed by Chen et al. [41], the pooled HR in ESCC for P21 was 1.28. However, one subsequent original study has confirmed that P21 expression was an independent favorable prognostic factor in ESCC [12].

#### P27

Chen et al. [41] also showed that the pooled HR in ESCC for P27 was 0.51, indicating that P27 was an independent favourable prognostic factor in ESCC.

### Associations between growth suppression-related markers and prognosis of ESCC patients

Retinoblastoma-associated protein (Rb) and P53 are two prototypical tumor suppressors that have been hotspots of prognostic marker research for many years (Tables 1, 3).

#### Rb

The prognostic significance of Rb in ESCC has been studied by multiple groups [11, 44, 45, 47–51]. However, only 1 original study reported the association between Rb expression and favorable prognosis with univariate analysis [48].

#### P53

Chen et al. [41] systematically reviewed 20 original studies concerning the relationship between P53 expression and the prognosis of ESCC, and revealed that P53 expression was an unfavorable prognostic marker. However, the pooled HR in ESCC for P53 was close to 1. There were five subsequent original studies [20, 30, 52–54], only one of which showed independent prognostic significance of P53 in ESCC [30].

### Associations between apoptosis-related markers and prognosis of ESCC patients

Seven markers function as regulators of apoptosis, including murine double minute gene 2 (MDM2), Survivin, Fas, Bax, Bcl-2, Bcl-x, and Caspase-3 (Tables 1, 4).

#### MDM2

The independent prognostic significance of MDM2 expression for patients with ESCC was determined in 2 large original studies [55, 56]. Another study demonstrated that MDM2 expression was an independent prognostic factor exclusively in the p53-negative subgroup [57]. Three reports claimed no association [11, 20, 49].

#### Survivin

Two meta-analyses demonstrated that Survivin was an independent unfavorable prognostic factor in ESCC with significant heterogeneity [58, 59]. Li et al. [58] further indicated that Survivin expression in the nuclei had an unfavorable impact on ESCC patient survival, whereas Survivin expression in the cytoplasm has no prognostic significance. Chen et al. [41] showed that the pooled HR of Survivin expression estimated for survival was 1.57, but the 95% CI covered 1.00.

#### Fas

The independent prognostic significance of Fas for a favorable outcome of ESCC was demonstrated in 2 original studies [60, 61], but was not confirmed in 2 other original studies [62, 63].

#### Bax

Three of 9 original studies demonstrated the prognostic value of Bax for a good outcome with univariate analysis or log-rank test [13, 45, 64], with 2 original studies demonstrating statistical significance with multivariate analysis [45, 64]. Only one original study of ESCC patients treated with neochemotherapy reported that Bax expression was associated with unfavorable prognosis [65]. No association were identified between Bax expression and clinical outcome of ESCC patients in other studies [62, 66–69]. This discrepancy may be due to the different treatments employed.

#### Bcl-2 and Bcl-x

Original studies of the prognostic role of Bcl-2 and Bcl-x in ESCC yielded conflicting results. Most original studies revealed that Bcl-2 or Bcl-x expression had no impact on the clinical outcome of patients with ESCC [63, 65, 66]. The independent prognostic value of Bcl-2 and Bcl-x expression was verified in one study each [62, 67]. Contrasting conclusions were also drawn in other original studies [45, 70].

#### Caspase-3

The largest original study suggested that Caspase-3 expression may be an independent prognostic indicator for primary resectable ESCC [21]. Consistently, Jiang et al. [14] reported that the up-regulation of Caspase-3 expression was associated with favorable prognosis. However, no independent prognostic significance of Caspase-3 in ESCC was elucidated in 2 other original studies [62, 65].

### Associations between angiogenesis-related markers and prognosis of ESCC patients

The prognostic values of vascular endothelial growth factor (VEGF) and hypoxia-inducible factor-1α (HIF-1α), key regulators of angiogenesis, have been studied exhaustively in ESCC (Tables 1, 5).

#### VEGF

Two meta-analyses revealed the prognostic significance of elevated VEGF expression for poor prognosis among patients with ESCC [41, 71]. Four additional original studies also reported unfavorable prognosis for ESCC patients with VEGF overexpression [15, 52, 72, 73], with the prognostic significance confirmed by multivariate analysis in 2 original studies [15, 72].

#### HIF-1α

Two meta-analyses revealed a significant association of increased HIF-1α expression with unfavorable prognosis in ESCC [74, 75]. There were 2 additional original studies [76, 77] after the meta-analyses. Zhang et al. [77] confirmed the association of HIF-1α overexpression with poor prognosis in ESCC patients with log-rank test. Furthermore, they revealed that HIF-1α expression in tumor cells was an independent prognostic marker for patients with locoregional or metastatic ESCC with multivariate analysis.

### Associations between invasion- and metastasis-related markers and prognosis of ESCC patients

Multiple markers involved in activating invasion and metastasis are summarized, including E-cadherin, α-catenin, β-catenin, Podoplanin, Fascin, and metastasis-associated protein 1 (MTA1) (Tables 1, 6).

#### E-cadherin

Two research groups conducted meta-analyses to investigate the effect of E-cadherin on the prognosis of ESCC [41, 78]. One original study was involved in both meta-analyses, evaluating E-cadherin expression by enzyme-linked immunosorbent assay (ELISA) instead of IHC. Both meta-analyses suggested that reduced E-cadherin expression was a prognostic indicator for short survival in ESCC, although the 95% CI of pooled HR covers 1.00 in the analysis by Chen et al. [41]. One subsequent study also revealed the association between reduced E-cadherin expression and short survival using the log-rank test [79].

#### α-Catenin

Nakanishi et al. [22] reported that down-regulation of α-catenin was associated with poor prognosis in patients with ESCC using the log-rank test, but no statistical significant association was revealed in multivariate analysis. Setoyama et al. [80] demonstrated the independent favorable prognostic significance of α-catenin. Two other original studies revealed no prognostic value of α-catenin in ESCC [26, 81].

#### β-Catenin

Although β-catenin has been studied by many groups, its effect on the prognosis of ESCC remains inconclusive. Two original studies confirmed that β-catenin was an independent prognostic factor for short survival of ESCC patients [23, 82]. By contrast, Hsu et al. [83] reported that membranous β-catenin expression was associated with good prognosis independently, whereas cytoplasmic β-catenin expression was not associated with patient survival. Other original studies indicated that β-catenin had no effect on the outcome of patients with ESCC [26, 62, 81, 84–86].

#### Podoplanin

Podoplanin expression was independently associated with poor outcomes in patients with ESCC as consistently reported by 4 separate original studies [16, 87–89]. In one other study, high podoplanin expression was significantly associated unfavorite prognosis only in univariate analysis [90].

#### Fascin

Fascin overexpression independently predicted poor prognosis in ESCC patients in 3 separate original studies [29, 91, 92], but no association between Fascin expression and patient survival was identified in another study [93].

#### MTA1

Luo et al. [94] conducted a meta-analysis to examine the relationship between MTA1 and survival of patients with solid tumors. Three of the 4 involved original studies determined that MTA1 overexpression was associated with short survival of ESCC patients. The pooled HR of MTA1 overexpression in ESCC was 1.86, with no significant heterogeneity.

### Associations between energy metabolism-related markers and prognosis of ESCC patients

Pyruvate kinase M2 (PKM2) is involved in energy metabolism, whose prognostic value in ESCC was studied (Table 7).

#### PKM2

Four original studies consistently elucidated the prognostic value of PKM2 expression for poor clinical outcome [17, 24, 95, 96], with the prognostic significance confirmed by multivariate analysis in 3 original studies [17, 95, 96]. These findings provide evidence of the significance of PKM2 expression as a prognostic biomarker in ESCC.

### Associations between immune regulation-related markers and prognosis of ESCC patients

Three markers involved in immune regulation, programmed cell death-ligand 1 (PD-L1), C-X-C chemokine receptor type 4 (CXCR4), and cyclooxygenase-2 (COX-2), have been studied for their prognostic implications in ESCC (Tables 1, 7).

#### PD-L1

Qu et al. [97] performed a meta-analysis of the prognostic significance of PD-L1 expression in ESCC patients. The study showed that overexpression of PD-L1 tended to be associated with short OS in ESCC; however, the difference did not reach statistical significance (*P* = 0.07).

#### CXCR4

The expression of CXCR4 was an unfavorable independent prognostic factor in ESCC in one report [98]. An association of CXCR4 expression and survival was revealed by log-rank test in another 2 original studies, although statistical significance was not achieved in multivariate analysis [99, 100]. However, 2 other original studies claimed no association between CXCR4 expression and the prognosis of ESCC patients [25, 101].

#### COX-2

Li et al. [102] systematically reviewed 12 original studies analyzing the prognostic significance of COX-2 expression in ESCC published before December 2008. A quantitative meta-analysis revealed that COX-2 overexpression was significantly associated with short OS. Chen et al. [41] performed meta-analyses on 2 original studies involved in Li’s review [102] and 2 additional relative original studies published after 2008. COX-2 expression was marginally significant as a prognostic marker in ESCC [41]. Ten of 14 original studies enrolled in these meta-analyses revealed that high expression of COX-2 was associated with short survival. However, the prognostic significance was confirmed by multivariate analysis in only one study with more than 50 patients enrolled.

### Associations between other markers and prognosis of ESCC patients

#### Octamer-binding transcription factor 4 (OCT4)

Nagaraja et al. [103] systematically reviewed 4 original studies of OCT4 expression and the clinical outcome of patients with ESCC published before May 2013. Meta-analysis showed that the positive rate of OCT4 was 53.6%. The HR of OCT4 expression for poor prognosis was 2.9, indicating the unfavourable prognostic role of OCT4 in ESCC.

#### Mut-L-homologon-1 (MLH1)

Reduced MLH1 expression has been demonstrated to be an independent prognostic indicator for poor prognosis in ESCC [104]. Consistently, Uehara et al. [105] revealed that MLH1 expression was associated with favourable prognosis as determined using log-rank test; they further demonstrated that the combination of MLH1 and Mut-S-Homologon-2 (MSH2) expression was an independent prognostic indicator as determined using multivariate analysis. However, no significant association between MLH1 expression and patient survival was identified in another study [18].

## Discussion

In this review, we summarized that 8 markers (EGFR, p-mTOR, Cyclin D1, Survivin, VEGF, Podoplanin, Fascin, and PKM2) were associated with poor prognosis and 3 markers (P27, P16, E-cadherin) were associated with good prognosis of ESCC (Additional file 3: Table S3). All these markers were investigated by 4 or more groups. More than half of the original studies revealed that the expression of the given protein was significantly associated with prognosis. In addition, the independent prognostic significance of these markers was demonstrated by multivariate analysis in 3 or more original studies. The strong evidence above suggests that the prognostic significance of these markers warrants prospective confirmation in large, well-defined clinical trials. Moreover, the prognostic significance of HIF-1α, MTA1, and OCT4 has been delineated by meta-analyses. However, these proteins do not meet our criteria for “emerging markers”.

The prognostic values of several markers, such as P53, Rb, and HER2, in ESCC have been studied exhaustively. Studies that evaluated the impact of P53 expression on the outcome of ESCC patients have yielded conflicting results. A meta-analysis conducted by Chen et al. [41] showed that the pooled HR of P53 for prognosis is approximately 1. Although the prognostic values of Rb and HER2 were evaluated in 4 or more cohorts, no independent prognostic significance was demonstrated, indicating that their prognostic values are, at best, weak.

We have selected prognostic biomarkers based on strong evidence that may help guide clinical practice. Several studies demonstrated that ESCC patients with high EGFR expression showed a higher response rate to EGFR inhibitors and monoclonal antibodies against EGFR as well as longer PFS and/or OS than those with low to moderate EGFR expression [106–109], although controversial results have also been reported [110]. In addition, the predictive implication of the expression of VEGF and p-mTOR for bevacizumab or everolimus treatment of ESCC patients, respectively, merits further investigation. Although inhibitors of other prognostic markers have not been developed or applied in clinical practice yet, the status of these markers may help clinicians to choose between aggressive and conservative treatments. However, it remains a large challenge to translate these research results into clinical practice. As summarized by Ludwig and Weinstein [111], biomarkers should be validated in prospective, well-controlled clinical studies of diverse patient populations across multiple institutions with well-established standards for sample preparation, data capture, statistical analysis, and scoring. In IHC marker research, antibodies with high sensitivity and specificity are pivotal, and studies that identify the best scoring methods for each potential marker are warranted.

This systematic review is subject to limitations. We focused primarily on only the prognostic significance of individual markers in this review. Many studies have attempted to evaluate multiple markers simultaneously. In some of the studies, a panel of markers predicted prognosis, although individual markers exhibited no prognostic significance [11, 45]. Due to the wide variety of different combinations of markers, it is beyond the scope of the current review to summarize prognostic panels of markers. However, given the complexity of the transformation process, a panel of molecules involved in different pathways may be able to predict prognosis with higher sensitivity and specificity than individual markers. Therefore, marker panels with putative prognostic value should be generated based on emerging individual prognostic markers.

## Conclusions

Here we summarized 11 emerging prognostic markers in ESCC based on sufficient evidence in this systematic review that warrant validation in large prospective clinical trials. These markers might be useful in predicting prognosis and facilitating personalized therapy decision-making for ESCC patients.

## Additional files

**Additional file 1: Table S1.** Description of original studies included in the systematic review.

**Additional file 2: Table S2.** Assessment of prognostic biomarker studies for risk of bias using the “Quality Assessment in Prognostic studies” (QUIPS) tool.

**Additional file 3: Table S3.** Summary of the identified prognostic markers in ESCC.

## Abbreviations

COX2: cyclooxygenase-2
CXCR4: C-X-C chemokine receptor type 4
DFS: disease-free survival
DSS: disease-specific survival
EGFR: epidermal growth factor receptor
ELISA: enzyme-linked immunosorbent assay
ESCC: esophageal squamous cell carcinoma
HER2: human epidermal growth factor receptor-2
HIF-1α: hypoxia-inducible factor-1α
HR: hazard ratio
IHC: immunohistochemistry
MDM2: murine double minute gene 2
MLH1: mut-L-homologon-1
MTA1: metastasis-associated protein 1
MYBL2: MYB proto-oncogene like 2
mTOR: mammalian target of rapamycin
NOTCH1: neurogenic locus notch homolog protein 1
OCT4: octamer-binding transcription factor 4
OS: overall survival
PCR: polymerase chain reaction assay
PD-L1: programmed cell death ligand 1
PKM2: pyruvate kinase M2
Rb: retinoblastoma-associated protein
VEGF: vascular endothelial growth fac
