# Supplementary material for: Immunohistochemical prognostic markers of esophageal squamous cell carcinoma: a systematic review
Source: Chin J Cancer. 2017 Aug 17;36:65. doi: 10.1186/s40880-017-0232-5 (PMC5561640; doi:10.1186/s40880-017-0232-5)
Supplement: Supplementary file 1 — Additional file 1: Table S1. Description of original studies included in the systematic review. [file 40880_2017_232_MOESM1_ESM.docx]

| **Table S1. Description of original studies included in the systematic review.** | | | | | | | | | | |
| --- | --- | --- | --- | --- | --- | --- | --- | --- | --- | --- |
| **Reference** | **Year** | **Country** | **Involved markers** | **Sample size** | **Median age** | **Percentage of men (%)** | **Stage** | **Endpoint(s)** | **Analytic methods and additional results** | **Adjusting factors** |
| Zhang *et al.* [10] | 2014 | China | EGFR | 441 | - | 78 | IB-IIIC | OS, DFS | Cox proportional hazards model | Age, gender, T, N, grade, treatment |
| Mathew *et al.* [11] | 2002 | India | P16, Rb, MDM2 | 100 | - | 60 | I-IV | OS | Log-rank test: Cyclin D1+/P16-, *P* = 0.023; pRb-/P16-/P21-, *P* = 0.03; P53+/P16-/pRb-, *P* = 0.02 | - |
| Shiozaki *et al.* [12] | 2013 | Japan | P21 | 69 | - | 83 | I-IV | OS | Cox proportional hazards model | T, N, stage, histological type, lymphatic invasion, venous invasion |
| Ikeguchi *et al.* [13] | 2001 | Japan | Bax | 141 | - | - | 0-IV | DSS | Cox proportional hazards model, univariate analysis, *P* = 0.013 | Stage |
| Jiang *et al.* [14] | 2010 | China | Caspase-3 | 64 | - | 53 | 0-III | OS | Cox proportional hazards model, log-rank test: *P* = 0.007 | Stage, infiltration depth, N, grade |
| Tao *et al.* [15] | 2012 | China | VEGF | 90 | - | - | - | OS, DFS | Cox proportional hazards model | Age, gender, tumor site, gross tumor volume, tumor size, grade, invasion depth, stage, N, MMP-1 |
| Tong *et al.* [16] | 2012 | China | Podoplanin | 56 | - | 80 | I-IV | OS | Cox proportional hazards model | Stage |
| Zhan et al. [17] | 2013 | China | PKM2 | 210 | - | 77 | 0-IV | OS | Cox proportional hazards model | Age, gender, differentiation, stage |
| Tzao et al. [18] | 2005 | China | MLH1 | 60 | - | - | I-IV | OS | Log-rank test | - |
| Zhang *et al.* [19] | 2014 | China | EGFR, HER2, VEGF | 128 | 55.8 | 67 | II-IV | OS | Log-rank test | - |
| Okamoto *et al.* [20] | 2013 | Japan | P16, P53, MDM2 | 86 | 63.9 | 84 | I-IV | OS, DFS | Cox proportional hazards model | Age, gender, tumor location, histological type, T, N, stage, lymphatic invasion, venous invasion |
| Wang *et al.* [21] | 2014 | China | Caspase-3 | 122 | 58 | 84 | - | DSS | Cox proportional hazards model | Age, N |
| Nakanishi *et al.* [22] | 1997 | Japan | α-catenin | 96 | 61.7 | 92 | I-III | OS | Cox proportional hazards model | N, invasion depth, vascular invasion |
| Lv *et al.* [23] | 2012 | China | β-catenin | 70 | 60 | 73 | I-IV | OS | Log-rank test | - |
| Li *et al.* [24] | 2014 | China | PKM2 | 141 | 61 | 60 | I-IV | OS | Cox proportional hazards model, log-rank test: *P* < 0.001 | Tumor size, T, N, differentiation, stage |
| Gockel *et al.* [25] | 2006 | Germany | CXCR4 | 53 | 57 | 88 | I-III | OS | Log-rank test | - |
| Nair *et al.* [26] | 2006 | Durban | α-catenin, β-catenin | 100 | 56 | 56 | I-III | OS | Log-rank test | - |
| Cao *et al.* [29] | 2014 | China | EGFR, Fascin | 315 | 59 | 79 | I-IV | OS | Cox proportional hazards model (univariate), *P* = 0.580 (generation dataset), *P* = 0.038 (validation dataset) | Stage |
| Shang *et al.* [30] | 2014 | China | EGFR, P53 | 590 | 61 | 73 | I-III | OS | Cox proportional hazards model | Gender, tumor size, grade, T, N, stage |
| Jiang *et al.* [31] | 2015 | China | EGFR | 96 | 62 | 83 | - | DFS, OS | Log-rank test, DFS: *P* = 0.006; OS: *P* = 0.007 | - |
| Xu *et al.* [32] | 2014 | China | EGFR | 87 | 66 | 86 | I-III | OS | Cox proportional hazards model | T, N |
| Mimura *et al.* [33] | 2005 | Japan | HER2 | 66 | 65.3 | 94 | 0-IV | OS | Cox proportional hazards model, log-rank test, *P* = 0.05 | T, N |
| Sunpaweravong *et al.* [34] | 2005 | Thailand | HER2 | 55 | 63 | 73 | II-IV | OS | Log-rank test, *P* = 0.04 | - |
| Zhan *et al.* [35] | 2012 | China | HER2 | 145 | 59 | 81 | I-IV | OS | Log-rank test, *P* = 0.036 | - |
| Hirashima *et al.* [36] | 2010 | Japan | p-mTOR | 143 | 63.8 | 88 | I-III | OS | Cox proportional hazards model | T, N |
| Kim *et al.* [37] | 2013 | Korea | p-mTOR | 165 | 65 | 96 | I-IV | DFS, OS | Cox proportional hazards model, p-mTOR/mTOR: PFS, HR=1.95, *P* = 0.006; OS, HR=1.87, *P* = 0.014 | Age, stage, chemotherapy, radiotherapy |
| Li *et al.* [38] | 2015 | China | p-mTOR | 105 | 54 | 98 | I-IV | DFS, OS | Cox proportional hazards model. DFS, *P* = 0.014; OS, *P* = 0.022 | Stage, grade, resection margin |
| Li *et al.* [39] | 2012 | China | p-mTOR | 77 | 52 | 97 | II-III | DFS, OS | Cox proportional hazards model | T, N |
| Cao *et al.* [42] | 2014 | China | P16 | 105 | 60 | 80 | I-III | OS, PFS | Cox proportional hazards model | T, N, current smoker, hemoglobin, ECOG performance status |
| Guan et al. [43] | 2013 | China | P16 | 90 | 61 | 70 | I-IV | OS | Cox proportional hazards model | Age, gender, grade, tumor size, invasion depth, M |
| Takeuchi et al. [44] | 2004 | Japan | P16, Rb | 90 | 61 | 87 | I-III | OS | Multivariate regression analysis | Age, gender, T, N, metastatic lymph nodes |
| Guner et al. [45] | 2003 | Germany | P16, Rb, Bax, Bcl-2 | 53 | 54.9 | 75 | I-III | OS | Cox proportional hazards model: interaction of Cyclin D1*P16*Bax, HR = 5.42, P = 0.0028; univariate: P16, P = 0.0129 | T, N, stage, grade, age, gender, radiotherapy or chemoradiotherapy, surgical procedure, P53 mutation |
| Fujiwara *et al.* [46] | 2008 | Japan | P16 | 60 | 65 | 82 | I-IV | OS | Cox proportional hazards model (univariate) *P* = 0.048 | Age, gender, tumor size, grade, T, N, lymphatic invasion |
| Ikeguchi *et al.* [47] | 2000 | Japan | Rb | 191 | 65 | 90 | I-IV | OS | Cox proportional hazards model, P53-/pRb+, good prognosis, *P* = 0.042 | Stage, tumor size |
| Ikeguchi *et al.* [48] | 2002 | Japan | Rb | 107 | 64.2 | 90 | I-IV | DFS | Cox proportional hazards model, log-rank test: pRB+/Cyclin D1+, good prognosis, *P* = 0.001 | Stage |
| Nam *et al.* [49] | 2008 | Korea | Rb | 51 | 65 | 98 | I-IVa | DSS | Log-rank test | - |
| Nita *et al.* [50] | 1999 | Japan | Rb | 62 | 62.8 | 81 | I-III | OS | Log-rank test | - |
| Wang *et al.* [51] | 2012 | China | Rb, MDM2 | 100 | 59 | 78 | I-IV | OS | Log-rank test, Cox proportional hazards model: CCND1+/pRb/ppRb+, OR = 4.172, *P* = 0.013 | - |
| Huang *et al.* [52] | 2013 | China | P53 | 106 | 60 | 58 | I-IV | OS | Cox proportional hazards model | Age, gender, N, invasion depth |
| Murata *et al.* [53] | 2013 | Japan | P53, VEGF | 266 | 65 | - | I-IV | OS, DFS | Log-rank test | - |
| Wang *et al.* [54] | 2013 | China | P53 | 114 | 58.1 | 89 | II-III | OS | Cox proportional hazards model: P53/LC3A, HR = 2.8, *P* = 0.027 | - |
| Ikeguchi *et al.* [55] | 2002 | Japan | MDM2 | 148 | 64.2 | 90 | I-IV | DFS | Cox proportional hazards model | Stage, pRb |
| Sun *et al.* [56] | 2015 | China | MDM2 | 149 | 54.7 | 77 | I-IV | OS | Cox proportional hazards model, PPARG+MDM2+NANOG: cohort 1, HR = 3.204, *P* = 0.005; cohort 2, HR = 3.956, *P* = 0.025 | N |
| Cheng *et al.* [57] | 2009 | China | MDM2 | 119 | 64.9 | 96 | I-IV | OS | Cox proportional hazards model | T, N, M |
| Chan *et al.* [60] | 2006 | China | Fas | 58 | 63.5 | 83 | I-IV | OS | Cox proportional hazards model | Age, gender, differentiation, T, N, stage, R category of resection |
| Shibakita *et al.* [61] | 1999 | Japan | Fas | 106 | 63.1 | 91 | I-IV | DFS | Cox proportional hazards model | Stage, amount of blood transfusion, tumor size, lymph vessel invasion, blood vessel invasion, Borrmann classification |
| Chang *et al.* [62] | 2005 | Korea | Fas, Bax, Bcl-2, Caspase-3, β-catenin | 118 | 60 | 94 | I-IV | OS | Log-rank test | - |
| Takikita *et al.* [63] | 2009 | China | Fas, Bcl-2 | 313 | 58 | 66 | I-IV | OS | Cox proportional hazards model | Gender, age, tobacco use, alcohol use, family history of UGI cancer, tumor grade, tumor stage, metastasis, degree differentiation. |
| Sturm *et al.* [64] | 2001 | Germany | Bax | 53 | 54.9 | 75 | I-IV | OS | Cox proportional hazards model: Bax/P16, HR = 0.26, *P* = 0.0063 | N |
| Kurabayashi *et al.* [65] | 2001 | Japan | Bax, Bcl-2, Caspase-3 | 76 | 64 | 87 | 0-IVa | OS | Log-rank test | - |
| Natsugoe *et al.* [66] | 2001 | Japan | Bax, Bcl-x | 111 | 64 | 93 | II-IV | OS | Log-rank test | - |
| Takayama *et al.* [67] | 2001 | Japan | Bax, Bcl-2, Bcl-x | 86 | 62.5 | 74 | I-IV | OS | Cox proportional hazards model | T, N, grade |
| Matsumoto *et al.* [68] | 2001 | Japan | Bax, Bcl-x | 79 | 63.7 | 94 | - | OS | Cox proportional hazards model | Age, gender, invasion depth, N |
| Sarbia *et al.* [69] | 1997 | Germany | Bax | 172 | 58 | 77 | I-IV | OS | Log-rank test | - |
| Torzewski *et al.* [70] | 1998 | Germany | Bcl-x | 172 | 58 | 77 | I-IV | OS | Cox proportional hazards model, log-rank test: *P* = 0.0485 | T, N, grade |
| Hou *et al.* [72] | 2015 | China | VEGF | 483 | 56 | 72 | I-III | DMFS, OS | Cox proportional hazards model | Age, gender, tumor location, tumor length, grade, stage |
| Omoto *et al.* [73] | 2014 | Japan | VEGF | 119 | 65.3 | 91 | - | OS | Cox proportional hazards model, log-rank test: *P* = 0.0005 | T, N |
| Shirakawa *et al.* [76] | 2012 | Japan | HIF-1α | 229 | 63 | 90 | I-IV | OS, DFS | Log-rank test | - |
| Zhang *et al.* [77] | 2014 | China | HIF-1α | 136 | 62 | 82 | I-IV | OS, DFS | Cox proportional hazards model, OS, HR = 1.991, *P* = 0.033; DFS, HR = 1.938, *P* = 0.037 | Gender, grade, T, N, stage |
| Ozawa *et al.* [79] | 2015 | Japan | E-cadherin | 83 | 63.1 | 84 | I-IV | OS, DSS | Log-rank test, OS, *P* = 0.022; DSS, *P* = 0.003 | - |
| Setoyama *et al.* [80] | 2007 | Japan | α-catenin | 205 | 64 | 89 | I-IV | OS | Cox proportional hazards model | Stage, lymphatic invasion, venous invasion |
| Lin *et al.* [81] | 2004 | China | α-catenin, β-catenin | 62 | 54 | 79 | I-III | OS | Log-rank test | - |
| Situ *et al.* [82] | 2010 | China | β-catenin | 227 | 58 | 73 | T2-3N0M0 | OS | Cox proportional hazards model | T, grade |
| Hsu *et al.* [83] | 2008 | China | β-catenin | 68 | 65 | 97 | I-IV | OS | Cox proportional hazards model, log-rank test | T, N, M, P53 |
| Zhao *et al.*[84] | 2003 | China | β-catenin | 106 | 59 | 72 | I-IV | OS, DFS | Log-rank test | - |
| Li *et al.* [85] | 2009 | China | β-catenin | 128 | 65 | 96 | I-IV | OS | Log-rank test | - |
| Deng *et al.* [86] | 2015 | China | β-catenin | 265 | 62 | 71 | - |  | Log-rank test | - |
| Chao *et al.* [87] | 2012 | China | Podoplanin | 113 | 56 | 96 | II-IV | DFS, DSS | Cox proportional hazards model | CRM margin, LVI |
| Nakashima *et al.* [88] | 2013 | Japan | Podoplanin | 101 | 64 | 87 | I-IV | OS | Cox proportional hazards model | Histology, T, lymphatic invasion, venous invasion, LN |
| Tanaka *et al.* [89] | 2015 | Japan | Podoplanin | 139 | 63.4 | 92 | I-III | OS | Cox proportional hazards model | Age, gender, T, N |
| Rahadiani *et al.*[90] | 2010 | Japan | Podoplanin | 61 | 65 | 95 | I-IV | OS, DFS | Cox proportional hazards model, log-rank test, OS, *P* = 0.0164; DFS, *P* = 0.0161 | T, N, stage |
| Hashimoto *et al.* [91] | 2005 | Japan | Fascin | 200 | 65 | 86 | I-IV | OS | Cox proportional hazards model | Age, gender, N, M |
| Zhao *et al.* [92] | 2010 | China | Fascin | 254 | 55 | 75 | I-IV | OS | Cox proportional hazards model, p-Fascin: HR = 0.661, *P* = 0.03 | N |
| Takikita *et al.*[93] | 2011 | China | Fascin | 257 | 57 | 66 | I-IV | OS | Polytomous logistic regression | Age, gender |
| Zhang *et al.* [95] | 2013 | China | PKM2 | 86 | 65 | 74 | I-IV | OS | Cox proportional hazards model | Age, gender, tumor size, tumor differentiation, stage, N |
| Fukuda *et al.* [96] | 2015 | Japan | PKM2 | 31 | 65 | 85 | I-IV | OS | Cox proportional hazards model | T, N, differentiation, stage |
| Zhang *et al.* [98] | 2013 | China | CXCR4 | 136 | 62 | 82 | I-IV | DFS, OS | Cox proportional hazards model | Gender, WHO grade, T, N, TNM stage |
| Lu *et al.* [99] | 2011 | China | CXCR4 | 127 | 59 | 73 | I-III | DFS, OS | Cox proportional hazards model, log-rank test, OS, *P* = 0.020; DFS, *P* = 0.007 | Age, gender, tumor size, tumor depth, N, stage, grade |
| Qi *et al.* [100] | 2015 | China | CXCR4 | 60 | 63 | 82 | - | - | Log-rank test, *P* = 0.001 | - |
| Sasaki *et al.* [101] | 2009 | Japan | CXCR4 | 214 | 64.1 | 91 | I-IV | OS, DFS | Cox proportional hazards model | T, N, M, lymphatic invasion, venous invasion, gender |
| Kishi et al. [104] | 2003 | Japan | MLH1 | 156 | 59 | 84 | I-IV | DSS | Cox proportional hazards model | N, M, clinical response, histological response |
| Uehara et al. [105] | 2005 | Japan | MLH1 | 122 | 62.3 | 86 | I-IV | OS | Cox proportional hazards model | T, N, M, surgical margin |

EGFR: epidermal growth factor receptor; Rb: retinoblastoma-associated protein; MDM2: murine double minute gene 2; VEGF: vascular endothelial growth factor; HER2: human epidermal growth factor receptor-2; PKM2: pyruvate kinase M2; CXCR4: C-X-C chemokine receptor type 4; p-mTOR: phosphorylated mammalian target of rapamycin; HIF-1α: hypoxia-inducible factor-1α; MLH1: Mut-L-homologon-1; OS: overall survival; DFS: disease-free survival; DSS: disease-specific survival; PFS: progression-free survival; DMFS: distant metastasis-free survival; HR: Hazard ratio; CCND1: cyclin D1; OR: odds ratio; LC3A: light chain 3A; PPARG: peroxisome proliferator-activated receptor gamma; NANOG: nanog homeobox; T: T stage/classification; N: N stage/classification; M: M stage/classification; MMP-1: matrix metalloproteinase-1; ECOG: eastern cooperative oncology group; UGI: upper gastrointestinal; CRM: circumferential resection margin; LVI: lymphovascular invasion; WHO: world health organization ; grade: histological grade; stage: clinical stage; “-” means “no data”.
