# Supplementary material for: Immunohistochemical prognostic markers of esophageal squamous cell carcinoma: a systematic review
Source: Chin J Cancer. 2017 Aug 17;36:65. doi: 10.1186/s40880-017-0232-5 (PMC5561640; doi:10.1186/s40880-017-0232-5)
Supplement: Supplementary file 2 — Additional file 2: Table S2. Assessment of prognostic biomarker studies for risk of bias using the “Quality Assessment in Prognostic studies” (QUIPS) tool. [file 40880_2017_232_MOESM2_ESM.docx]

| **Table S2. Assessment of prognostic biomarker studies for risk of bias using the "Quality Assessment in Prognostic studies"(QUIPS) tool** | | | | | |
| --- | --- | --- | --- | --- | --- |
| **Reference** | **Risk of bias** | | | | |
|  | **Study Participation** | **Study Attrition** | **Prognostic Factor Measurement** | **Outcome Measurement** | **Statistical Analysis and Reporting** |
| Zhang *et al.* [10] | Low | Low | Low | Low | Low |
| Mathew *et al.* [11] | Low | High | Low | Low | Low |
| Shiozaki *et al.* [12] | Moderate | Low | Low | Low | Low |
| Ikeguchi *et al.* [13] | Low | Low | Low | Low | Low |
| Jiang *et al.* [14] | Moderate | Low | Low | Low | Low |
| Tao *et al.* [15] | Moderate | Low | Low | Low | Low |
| Tong *et al.* [16] | Moderate | Low | Low | Low | Low |
| Zhan *et al.* [17] | Low | Low | Low | Low | Low |
| Tzao *et al.* [18] | Moderate | Low | Low | Low | Moderate |
| Zhang *et al.* [19] | Low | Moderate | Low | Low | Moderate |
| Okamoto *et al.* [20] | Moderate | Moderate | Low | Low | Low |
| Wang *et al.* [21] | Low | Moderate | Low | Low | Low |
| Nakanishi *et al.* [22] | Moderate | Moderate | Low | Low | Low |
| Lv *et al.* [23] | Moderate | Moderate | Low | Low | Moderate |
| Li *et al.* [24] | Low | Moderate | Low | Low | Low |
| Gockel *et al.* [25] | Moderate | Moderate | Low | Low | Moderate |
| Nair *et al.* [26] | Low | High | Low | Low | Low |
| Cao *et al.* [29] | Low | Low | Low | Low | Low |
| Shang *et al.* [30] | Low | Low | Low | Low | Low |
| Jiang *et al.* [31] | Moderate | Low | Low | Low | Moderate |
| Xu *et al.* [32] | Moderate | Low | Low | Low | Low |
| Mimura *et al.* [33] | Moderate | Low | Low | Low | Low |
| Sunpaweravong *et al.* [34] | Moderate | Low | Low | Low | Moderate |
| Zhan *et al.* [35] | Low | Low | Low | Low | Moderate |
| Hirashima *et al.* [36] | Low | Low | Low | Low | Low |
| Kim *et al.* [37] | Low | Low | Low | Low | Low |
| Li *et al.* [38] | Low | Low | Low | Low | Low |
| Li *et al.* [39] | Moderate | Low | Low | Low | Low |
| Cao *et al.* [42] | Low | Low | Low | Low | Low |
| Guan *et al.* [43] | Moderate | Low | Low | Low | Low |
| Takeuchi *et al.* [44] | Moderate | Low | Low | Low | Low |
| Guner *et al.* [45] | Moderate | Low | Low | Low | Low |
| Fujiwara *et al.* [46] | Moderate | Low | Low | Low | Low |
| Ikeguchi *et al.* [47] | Low | Low | Low | Low | Low |
| Ikeguchi *et al.* [48] | Low | Low | Low | Low | Low |
| Nam *et al.* [49] | Moderate | Low | Low | Low | Moderate |
| Nita *et al.* [50] | Moderate | Low | Low | Low | Moderate |
| Wang *et al.* [51] | Low | Low | Low | Low | Low |
| Huang *et al.* [52] | Low | Low | Low | Low | Low |
| Murata *et al.* [53] | Low | Low | Low | Low | Moderate |
| Wang *et al.* [54] | Low | Low | Low | Low | Moderate |
| Ikeguchi *et al.* [55] | Low | Low | Low | Low | Low |
| Sun *et al.* [56] | Low | Low | Low | Low | Low |
| Cheng *et al.* [57] | Low | Low | Low | Low | Low |
| Chan *et al.* [60] | Moderate | Low | Low | Low | Low |
| Shibakita *et al.* [61] | Low | Low | Low | Low | Low |
| Chang *et al.* [62] | Low | Low | Low | Low | Moderate |
| Takikita *et al.* [63] | Low | Low | Low | Low | Low |
| Sturm *et al.* [64] | Moderate | Low | Low | Low | Low |
| Kurabayashi *et al.* [65] | Moderate | Low | Low | Low | Moderate |
| Natsugoe *et al.* [66] | Low | Low | Low | Low | Moderate |
| Takayama *et al.* [67] | Moderate | Low | Low | Low | Low |
| Matsumoto *et al.* [68] | Moderate | Low | Low | Low | Low |
| Sarbia *et al.* [69] | Low | Low | Low | Low | Moderate |
| Torzewski *et al.* [70] | Low | Low | Low | Low | Low |
| Hou *et al.* [72] | Low | Low | Low | Low | Low |
| Omoto *et al.* [73] | Low | Low | Low | Low | Low |
| Shirakawa *et al.* [76] | Low | Low | Low | Low | Moderate |
| Zhang *et al.* [77] | Low | Low | Low | Low | Low |
| Ozawa *et al.* [79] | Moderate | Low | Low | Low | Low |
| Setoyama *et al.* [80] | Low | Low | Low | Low | Low |
| Lin *et al.* [81] | Moderate | Low | Low | Low | Moderate |
| Situ *et al.* [82] | Low | Low | Low | Low | Low |
| Hsu *et al.* [83] | Moderate | Low | Low | Low | Moderate |
| Zhao *et al.* [84] | Low | Low | Low | Low | Moderate |
| Li *et al.* [85] | Low | Low | Low | Low | Moderate |
| Deng *et al.* [86] | Low | Low | Low | Low | Moderate |
| Chao *et al.* [87] | Low | Low | Low | Low | Low |
| Nakashima *et al.* [88] | Low | Low | Low | Low | Low |
| Tanaka *et al.* [89] | Low | Low | Low | Low | Low |
| Rahadiani *et al.* [90] | Moderate | Low | Low | Low | Low |
| Hashimoto *et al.* [91] | Low | Low | Low | Low | Low |
| Zhao *et al.* [92] | Low | Low | Low | Low | Low |
| Takikita *et al.* [93] | Low | Low | Low | Low | Moderate |
| Zhang *et al.* [95] | Moderate | Low | Low | Low | Low |
| Fukuda *et al.* [96] | Low | Low | Low | Low | Low |
| Zhang *et al.* [98] | Low | Low | Low | Low | Low |
| Lu *et al.* [99] | Low | Low | Low | Low | Low |
| Qi *et al.* [100] | Moderate | Low | Low | Low | Moderate |
| Sasaki *et al.* [101] | Low | Low | Low | Low | Low |
| Kishi *et al.* [104] | Low | Low | Low | Low | Moderate |
| Uehara *et al.* [105] | Moderate | Low | Low | Low | Moderate |

The Study Participation domain addresses the representativeness of the study sample. The Study Attrition domain addresses whether participants with follow-up data represent persons enrolled in the study. The Prognostic Factor Measurement domain addresses adequacy of prognostic factor measurement. The Outcome Measurement domain addresses the adequacy of outcome measurement. The Statistical Analysis and Reporting domain addresses the appropriateness of the study’s statistical analysis and completeness of reporting.
