# Supplementary material for: Immunohistochemical prognostic markers of esophageal squamous cell carcinoma: a systematic review
Source: Chin J Cancer. 2017 Aug 17;36:65. doi: 10.1186/s40880-017-0232-5 (PMC5561640; doi:10.1186/s40880-017-0232-5)
Supplement: Supplementary file 3 — Additional file 3: Table S3. Summary of the identified prognostic markers in ESCC. [file 40880_2017_232_MOESM3_ESM.docx]

**Table S3. Summary of the identified prognostic markers in ESCC.**

| Marker | HR from meta-analyses | HR from original studies | Total number of supporting studies |
| --- | --- | --- | --- |
| EGFR | 1.600-1.768 | - | 14 |
| Cyclin D1 | 1.78-1.82 | - | 14 |
| VEGF | 1.81-1.84 | - | 29 |
| Survivin | 1.57-1.89 | - | 7 |
| P27 | 0.507 | - | 6 |
| E-cadherin | 0.72-0.81 | - | 10 |
| Podoplanin | - | 1.818-8.039 | 5 |
| Fascin | - | 1.56-1.79 | 4 |
| p-mTOR | - | 1.47-2.92 | 4 |
| PKM2 | - | 1.214-2.358 | 4 |
| P16 | - | 0.234-0.420 | 5 |

ESCC: esophageal squamous cell carcinoma; EGFR: epidermal growth factor receptor; VEGF: vascular endothelial growth factor; mTOR: mammalian target of rapamycin; PKM2: pyruvate kinase M2; HR: hazard ratio; -: no data.
